# Aberrant DNA methylation of M1-macrophage genes in coronary artery disease

**DOI:** 10.1038/s41598-018-38040-1

**Published:** 2019-02-05

**Authors:** Chetan Bakshi, Rajesh Vijayvergiya, Veena Dhawan

**Affiliations:** 1 Department of Experimental Medicine and Biotechnology, Postgraduate Institute of Medical Education and Research, Chandigarh, 160012 India; 2 Department of Cardiology, Postgraduate Institute of Medical Education and Research, Chandigarh, 160012 India

## Abstract

M1 and M2 macrophage balance in atherosclerosis has attracted much interest. Though, it remains unknown how macrophage heterogeneity is regulated. Moreover, the regulation of macrophage polarization and activation also involve DNA methylation. However, it remains ambiguous which genes are under direct regulation by DNA methylation. Our aim was to evaluate the gene-specific promoter DNA methylation status of M1/M2 polarization markers in PBMCs of CAD patients. A case-control study was performed with 25 CAD patients and 25 controls to study the promoter DNA methylation status of *STAT1*, *STAT6*, *MHC2*, *IL12b*, *iNOS*, *JAK1*, *JAK2* and *SOCS5* using MS-HRM analysis. Our data indicates that there was a clear-cut difference in the pattern of gene-specific promoter DNA methylation of CAD patients in comparison to controls. A significant difference was observed between the percentage methylation of *STAT1*, *IL12b*, *MHC2*, *iNOS*, *JAK1* and *JAK2* in CAD patients and control subjects. In conclusion, our data show that MS-HRM assay is a rapid and inexpensive method for qualitatively identifying aberrant gene-specific promoter DNA methylation changes in CAD. Furthermore, we propose that gene-specific promoter DNA methylation based on monocyte/macrophage might aid as diagnostic marker for clinical application or DNA methylation-related drug interventions may offer novel possibilities for atherosclerotic disease management.

## Introduction

Coronary artery disease (CAD), a most common cardiovascular disease (CVD), is a foremost cause of mortality globally^1^. The underlying pathological cause of CAD is atherosclerosis, a chronic inflammatory disease with cumulative deposition of lipoproteins in the coronary arteries eventually leading to impaired or no blood supply to the heart and myocardial infarction (MI)^2,3^. Atherosclerosis is a complex multi-factorial disease with numerous genetic and non-genetic risk factors^4^. Cells of the innate immunity, mostly monocytes recruited by the dysfunctional endothelium and monocyte-derived macrophages in the plaque, play an essential role in the initiation, progression, and eventual rupture of atherosclerotic lesions. In response to micro-environmental stimuli in the plaque, monocytes can differentiate into two distinct subsets of macrophages as either classical M1 profile with pro-inflammatory (killing) activity or alternative M2 profile with anti-inflammatory (repairing) activity^5,6^. However, recently other plaque-specific macrophage phenotypes have been adopted into the current paradigm^7–9^. Macrophage heterogeneity within atherosclerotic lesions has attracted much interest owing to the importance of balance between M1 and M2 population in determining the plaque outcome and its possible therapeutic implications^10,11^. However, it remains unknown how macrophage heterogeneity is regulated and its contribution in the initiation and propagation of atherosclerosis.

Activation of several interconnected pathways tightly regulates macrophage polarization and functions. Among all, the balance between activation of signal transducer and activator of transcription (STAT) 1 and STAT3/STAT6 transcription factors play a vital role^6,12^. STAT1 plays a crucial role in M1 macrophage polarization in the presence of interferon (IFN) γ. Stimulation of the IFNγ receptor initiates Janus kinase (JAK) 1- and JAK2-mediated signaling cascades, resulting in activation and binding of STAT1 as a homodimer to *cis* elements known as gamma-activated sequences in the promoters of the genes encoding inducible nitric oxide synthase (iNOS/NOS2), the major histocompatibility complex (MHC) class II transactivator (CIITA) and IL-12^13,14^. On the contrary, STAT6 is necessary for M2 macrophage polarization in the presence of interleukin (IL) −4 and/or IL-13^14^. After binding to its receptor (IL-4R), IL-4 starts JAK1- and JAK3-mediated intracellular cascade that culminates in the activation of STAT6, which then regulates transcription of its target genes, including arginase 1, macrophage mannose receptor 1, resistin-like-α and chitinase 3-like 3^13,15^.

The suppressor of cytokine signaling (SOCS) family of proteins can terminate adaptive and innate immune responses by functioning as feedback inhibitors of the JAK/STAT signaling pathways^16^. There are 8 SOCS proteins known to present in mammals; SOCS1-7 and the alternatively named cytokine-inducible SH2-containing protein (CISH)^16^. As a consequence of their regulatory properties, particularly due to their tumor suppressor and anti-inflammatory functions, SOCS proteins have gained recognition for their role in various diseases including CVDs. SOCS5 is known to be expressed in different adult tissues with highest expression in lymphoid organs, including the lymph nodes, spleen, bone marrow and thymus, with particular expression in primary B and T cells, which suggests they have potential immune-related functions^17,18^.

Nowadays, the influence of epigenetic modifications in cardiovascular pathophysiology is evolving as a crucial interface governing genotype to phenotype variability. Epigenetic mechanisms regulate varied cellular functions including cellular differentiation, cell activation and transformation. Moreover, the regulation of macrophage development, polarization and activation also involve deoxyribonucleic acid (DNA) methylation and post-translational histone modifications. DNA methylation is a mechanism which involves the addition of a methyl group to CpG dinucleotide nearby gene promoters, CpG shores and gene bodies, thereby causing transcriptional repression^19–21^. Both positive and negative correlations between CAD and global DNA methylation of long interspersed nuclear element-1 (LINE-1) and ALU repeats have been reported in the literature^22–26^. However, till date, most investigations have primarily focused on post-translational histone modifications and the role of epigenetic mechanisms, particularly DNA methylation needs to be further defined in cardiovascular pathophysiology.

Therefore, the current study aimed to 1) assess the gene-specific promoter DNA methylation status of established M1- and M2-associated macrophage polarization markers *STAT1*, *STAT6*, *MHC2*, *IL12b*, *iNOS*, *JAK1*, *JAK2* and *SOCS5* in peripheral blood mononuclear cells (PBMCs) of patients with CAD, and 2) subsequently compare the DNA methylation status of these genes in PBMCs of CAD patients and healthy controls to explore the role of DNA methylation in macrophage polarization.

## Results

### Genomic DNA isolation and bisulfite conversion of isolated DNA

**Figure 1:**
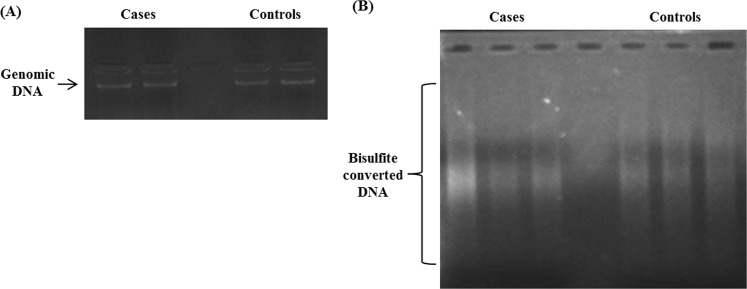
(**A**) Agarose gel electrophoresis of genomic DNA isolated from PBMCs of the study subjects. Lane 1 and 2 represent genomic DNA sample from CAD patients. Lane 4 and 5 represent genomic DNA sample from control subjects. 200 ng of genomic DNA was loaded in each well. (**B**) Agarose gel electrophoresis of bisulphite-converted genomic DNA. Lane 1, 2 and 3 represent bisulphite-modified DNA of CAD patients. Lane 5, 6 and 7 represent bisulphite-modified DNA of control subjects.

The integrity and purity of isolated genomic DNA was checked by agarose gel electrophoresis. We observed a single band of DNA on 0.8% agarose gel with no smearing, suggesting that the isolated DNA was intact and of good quality (Fig. 1A).

Isolated genomic DNA was then subjected to bisulfite conversion. On 1.5% agarose gel, of bisulfite-converted genomic DNA samples showed smearing pattern (Fig. 1B) as compared to unconverted genomic DNA (Fig. 1A). Bisulfite treatment of genomic DNA leads to fragmentation of genomic DNA due to the presence of endonucleases in bisulphite mix, thus resulting in appearance of smearing pattern when applied on agarose gel.

### Methylation sensitive-high resolution melting (MS-HRM) assay optimization

**Figure 2:**
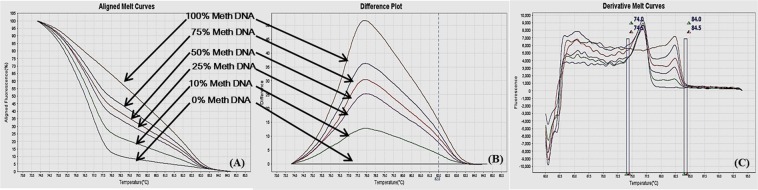
MS-HRM standard curves of STAT1. (**A**) Aligned melt curves, (**B**) Difference plot & (**C**) Derivative melt curves of 100%, 75%, 50%, 25%, 10% and 0% methylated DNA standards, normalized to the 0% methylated DNA. Aligned melt curves (**A**) show that the 100% methylated DNA had higher melting temperature as compared to unmethylated DNA for each gene analyzed by MS-HRM. The 75%, 50%, 25% and 10% methylated DNA standards had a melting temperature situated between the 100% and unmethylated DNA standards which is respective of the percentage methylation of sample. The graph of the negative first derivative of the melting curve (**C**) shows that the 100% methylated and unmethylated DNA had only one melt peak, whereas 75%, 50%, 25% and 10% standards had two melt peaks representative of each of the DNA standard.

**Figure 3:**
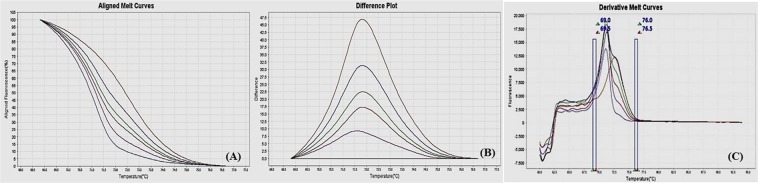
MS-HRM standard curves of STAT6. (**A**) Aligned melt curves. (**B**) Difference plot & (**C**) Derivative melt curves of 100%, 75%, 50%, 25%, 10% and 0% methylated DNA standards, normalized to the 0% methylated DNA. Aligned melt curves (**A**) show that the 100% methylated DNA had higher melting temperature as compared to unmethylated DNA for each gene analyzed by MS-HRM. The 75%, 50%, 25% and 10% methylated DNA standards had a melting temperature situated between the 100% and unmethylated DNA standards which is respective of the percentage methylation of sample. The graph of the negative first derivative of the melting curve (**C**) shows that the 100% methylated and unmethylated DNA had only one melt peak, whereas 75%, 50%, 25% and 10% standards had two melt peaks representative of each of the DNA standard.

**Figure 4:**
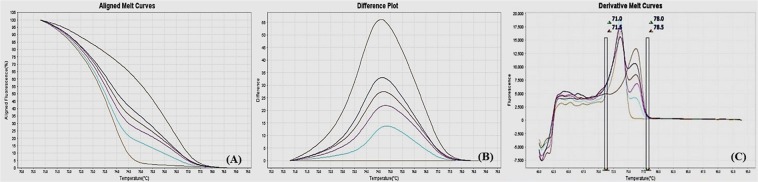
MS-HRM standard curves of IL12b, (**A**) Aligned melt curves, (**B**) Difference plot & (**C**) Derivative melt curves of 100%, 75%, 50%, 25%, 10% and 0% methylated DNA standards, normalized to the 0% methylated DNA. Aligned melt curves (**A**) show that the 100% methylated DNA had higher melting temperature as compared to unmethylated DNA for each gene analyzed by MS-HRM. The 75%, 50%, 25% and 10% methylated DNA standards had a melting temperature situated between the 100% and unmethylated DNA standards which is respective of the percentage methylation of sample. The graph of the negative first derivative of the melting curve (**C**) shows that the 100% methylated and unmethylated DNA had only one melt peak, whereas 75%, 50%, 25% and 10% standards had two melt peaks representative of each of the DNA standard.

**Figure 5:**
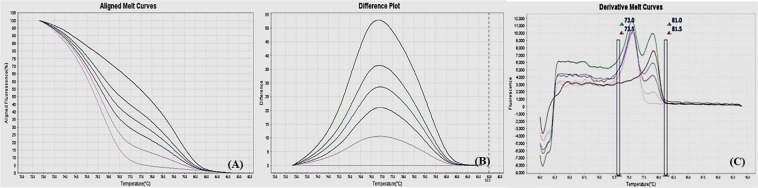
MS-HRM standard curves of MHC2, (**A**) Aligned melt curves, (**B**) Difference plot & (**C**) Derivative melt curves of 100%, 75%, 50%, 25%, 10% and 0% methylated DNA standards, normalized to the 0% methylated DNA. Aligned melt curves (**A**) show that the 100% methylated DNA had higher melting temperature as compared to unmethylated DNA for each gene analyzed by MS-HRM. The 75%, 50%, 25% and 10% methylated DNA standards had a melting temperature situated between the 100% and unmethylated DNA standards which is respective of the percentage methylation of sample. The graph of the negative first derivative of the melting curve (**C**) shows that the 100% methylated and unmethylated DNA had only one melt peak, whereas 75%, 50%, 25% and 10% standards had two melt peaks representative of each of the DNA standard.

**Figure 6:**
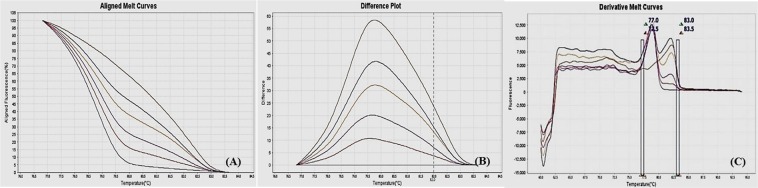
MS-HRM standard curves of iNOS. (**A**) Aligned melt curves. (**B**) Difference plot & (**C**) Derivative melt curves of 100%, 75%, 50%, 25%, 10% and 0% methylated DNA standards, normalized to the 0% methylated DNA. Aligned melt curves (**A**) show that the 100% methylated DNA had higher melting temperature as compared to unmethylated DNA for each gene analyzed by MS-HRM. The 75%, 50%, 25% and 10% methylated DNA standards had a melting temperature situated between the 100% and unmethylated DNA standards which is respective of the percentage methylation of sample. The graph of the negative first derivative of the melting curve (**C**) shows that the 100% methylated and unmethylated DNA had only one melt peak, whereas 75%, 50%, 25% and 10% standards had two melt peaks representative of each of the DNA standard.

**Figure 7:**
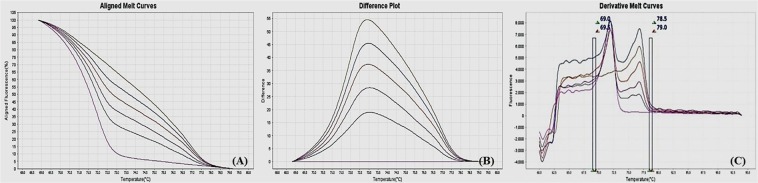
MS-HRM standard curves of SOCS5. (**A**) Aligned melt curves. (**B**) Difference plot & (**C**) Derivative melt curves of 100%, 75%, 50%, 25%, 10% and 0% methylated DNA standards, normalized to the 0% methylated DNA. Aligned melt curves (**A**) show that the 100% methylated DNA had higher melting temperature as compared to unmethylated DNA for each gene analyzed by MS-HRM. The 75%, 50%, 25% and 10% methylated DNA standards had a melting temperature situated between the 100% and unmethylated DNA standards which is respective of the percentage methylation of sample. The graph of the negative first derivative of the melting curve (**C**) shows that the 100% methylated and unmethylated DNA had only one melt peak, whereas 75%, 50%, 25% and 10% standards had two melt peaks representative of each of the DNA standard.

**Figure 8:**
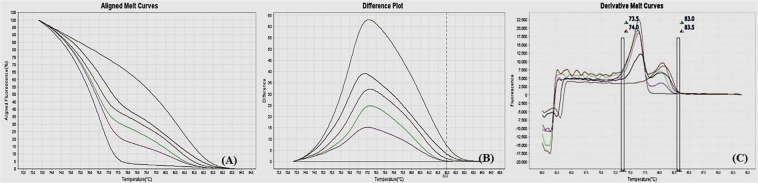
MS-HRM standard curves of JAK1. (**A**) Aligned melt curves. (**B**) Difference plot & (**C**) Derivative melt curves of 100%, 75%, 50%, 25%, 10% and 0% methylated DNA standards, normalized to the 0% methylated DNA. Aligned melt curves (**A**) show that the 100% methylated DNA had higher melting temperature as compared to unmethylated DNA for each gene analyzed by MS-HRM. The 75%, 50%, 25% and 10% methylated DNA standards had a melting temperature situated between the 100% and unmethylated DNA standards which is respective of the percentage methylation of sample. The graph of the negative first derivative of the melting curve (**C**) shows that the 100% methylated and unmethylated DNA had only one melt peak, whereas 75%, 50%, 25% and 10% standards had two melt peaks representative of each of the DNA standard.

**Figure 9:**
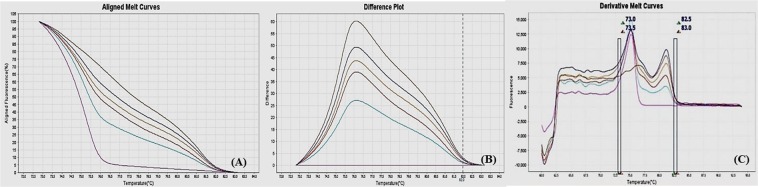
MS-HRM standard curves of JAK2 promoter region. (**A**) Aligned melt curves, (**B**) Difference plot & (**C**) Derivative melt curves of 100%, 75%, 50%, 25%, 10% and 0% methylated DNA standards, normalized to the 0% methylated DNA. Aligned melt curves (**A**) show that the 100% methylated DNA had higher melting temperature as compared to unmethylated DNA for each gene analyzed by MS-HRM. The 75%, 50%, 25% and 10% methylated DNA standards had a melting temperature situated between the 100% and unmethylated DNA standards which is respective of the percentage methylation of sample. The graph of the negative first derivative of the melting curve (**C**) shows that the 100% methylated and unmethylated DNA had only one melt peak, whereas 75%, 50%, 25% and 10% standards had two melt peaks representative of each of the DNA standard.

Different ratios of 100% methylated DNA and unmethylated DNA were assayed for optimization of the conditions, standard curve preparation and assessment of the analytical specificity and sensitivity of the MS-HRM assay. Fully methylated DNA will remain cytosine-rich after bisulphite conversion, whereas less- or un-methylated DNA will comprise a lesser GC-content due to conversion of unmethylated cytosines into uracil. Due to differences in the GC content, sequences result in melting temperature differences. The resultant data was analyzed by the HRM Software v2.0 (Applied Biosystems, Life Technologies) to generate aligned melt curves, difference plot and derivative melt curves of each standard for respective gene (Figs 2–9). In detail:

#### Annealing temperature

A range of annealing temperatures were tested for each gene. The temperature, at which the methylated DNA standards were best distinguishable from each other, was selected on the basis of normalized aligned melt curves and derivative plot.

#### Analytical specificity

The MS-HRM assay standardized for gene-specific promoter DNA methylation was highly specific for bisulfite converted DNA since under the experimental settings only bisulfite converted DNA was amplified. When unmethylated unconverted human genomic DNA was used as a negative control, amplification under the similar conditions was not observed. The negative first derivative graph of the melt curve showed that the 100% methylated and unmethylated DNA had only single melt peak each, on the other hand the standards with methylation levels in between had two melting peaks representative of respective DNA standard (Figs 2–9(C)). In addition, bisulfite converted 100% methylated and unmethylated controls could be readily discriminated and no “non-specific” products were observed.

#### Analytical sensitivity

The analytical sensitivity of the assay was evaluated by analysing the different dilutions of fully methylated to fully unmethylated DNA (0%, 10%, 25%, 50%, 75% and 100%). As expected, aligned melt curves showed that the 100% methylated DNA had higher melt temperature as compared to unmethylated DNA for each gene analyzed by MS-HRM. The 50% methylated DNA standard had melting temperature situated between the 100% and unmethylated DNA standards. Similarly, the other methylated standards had melting temperature situated according to their methylation levels (Figs 2–9(A)). The data also suggested that the standards with defined methylation levels (0–100%) were efficiently resolved by this method and there was no polymerase chain reaction (PCR) bias between the unmethylated and methylated DNA standards.

### Gene-specific promoter region CpG methylation by MS-HRM

CpG dinucleotides are generally located in clusters in human genome in CpG islands. Gene promoter associated CpG island methylation is usually associated with transcriptional repression due to binding of methyl-CpG binding proteins, which further recruit proteins, thereby blocking transcription. HRM analysis is used to differentiate single nucleotide differences on basis of the melting temperature of amplicons^27–30^.

**Figure 10:**
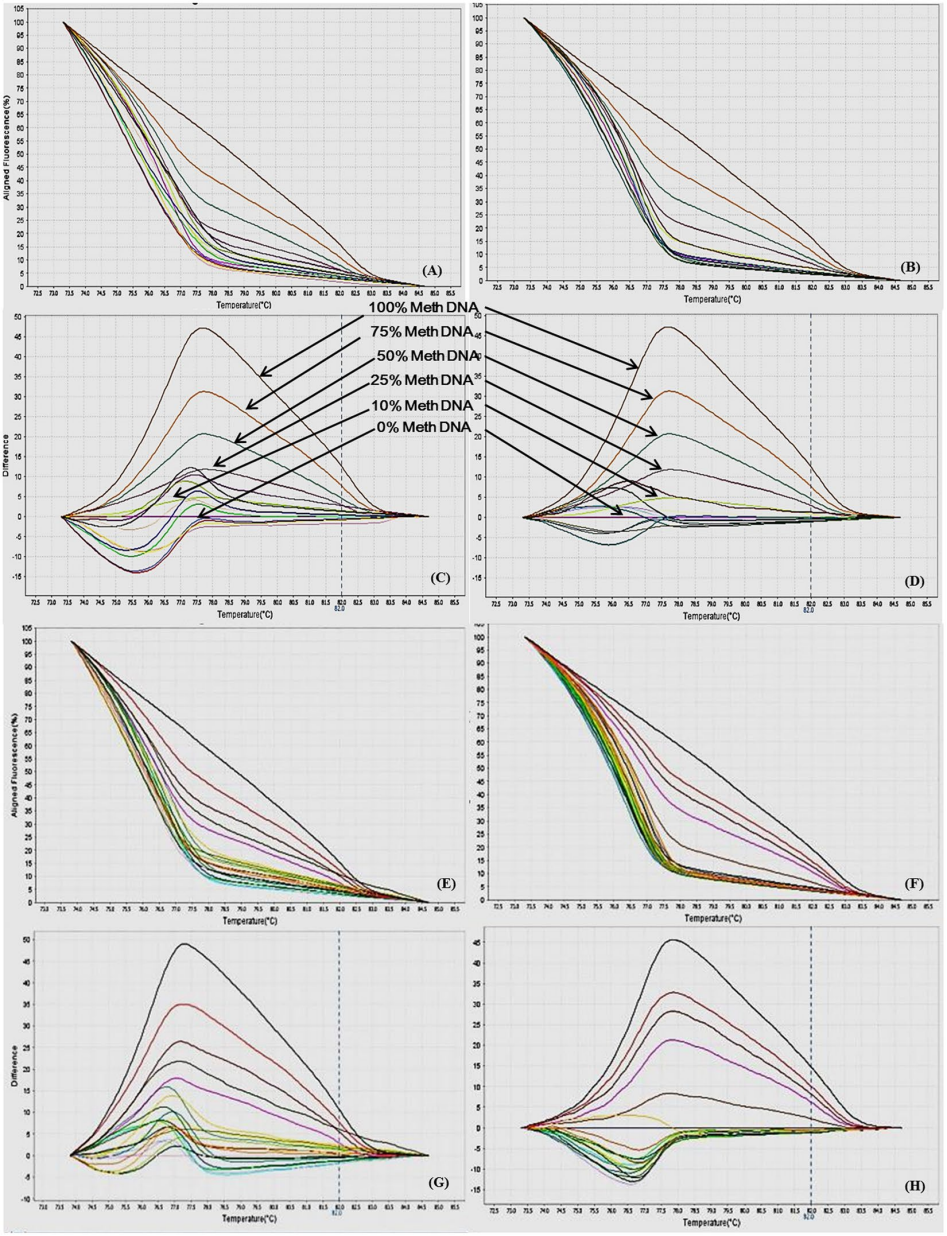
MS-HRM analysis for STAT1. (**A**,**B**) Aligned melt curves and (**C**,**D**) Difference plot of 100%, 75%, 50%, 25%, 10% and 0% methylated standards, CAD patient samples (**A**,**C**, n=10) and control samples (**B**,**D**, n = 10) normalized to the 0% methylated standard DNA, showing the differential fluorescence among few representative samples of all the categories included in the study. All amplicons from the samples form a clump in the low methylation region and begin melting before the unmethylated control as can be seen by the earlier drop in fluorescence resulting from heterogeneous methylation due to heteroduplex formation.  Each sample was analyzed in duplicate per reaction. (**E**,**F**) Aligned melt curves and (**G**,**H**) Difference plot of 100%, 75%, 50%, 25%, 10%, 0% methylated standards, CAD patient samples (**E**,**G**, n = 15) and control samples (**F**,**H**, n = 15) normalized to the 0% methylated standard DNA, showing the differential fluorescence of the remaining samples. All amplicons from the samples form a clump in the low methylation region and begin melting before the unmethylated control as can be seen by the earlier drop in fluorescence. Each sample was analyzed in duplicate per reaction.

**Figure 11:**
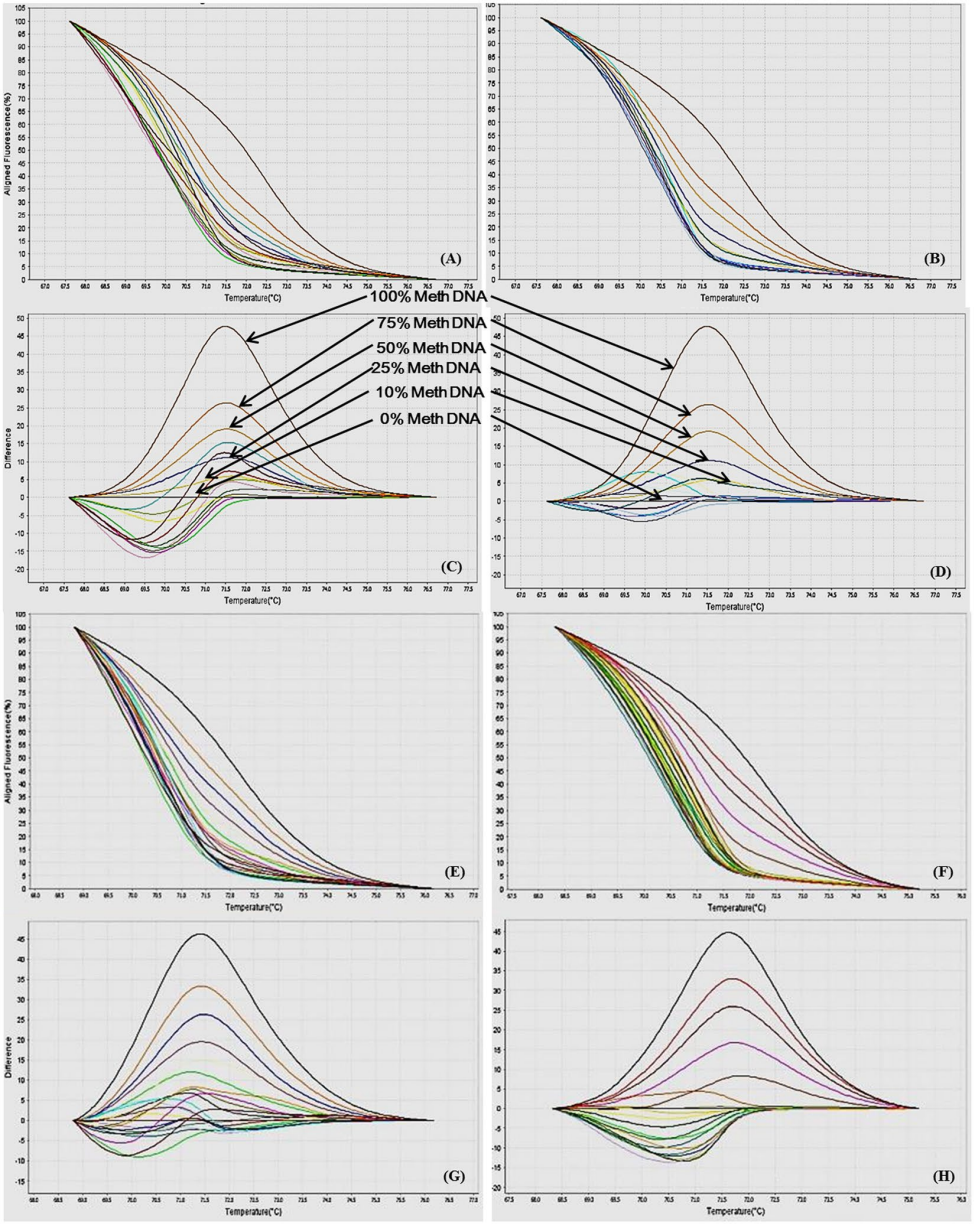
MS-HRM analysis for STAT6. (**A**,**B**) Aligned melt curves and (**C**,**D**) Difference plot of 100%, 75%, 50%, 25%, 10% and 0% methylated standards, CAD patient samples (**A**,**C**, n=10) and control samples (**B**,**D**, n = 10) normalized to the 0% methylated standard DNA, showing the differential fluorescence among few representative samples of all the categories included in the study. All amplicons from the samples form a clump in the low methylation region and begin melting before the unmethylated control as can be seen by the earlier drop in fluorescence resulting from heterogeneous methylation due to heteroduplex formation.  Each sample was analyzed in duplicate per reaction. (**E**,**F**) Aligned melt curves and (**G**,**H**) Difference plot of 100%, 75%, 50%, 25%, 10%, 0% methylated standards, CAD patient samples (**E**,**G**, n = 15) and control samples (**F**,**H**, n = 15) normalized to the 0% methylated standard DNA, showing the differential fluorescence of the remaining samples. All amplicons from the samples form a clump in the low methylation region and begin melting before the unmethylated control as can be seen by the earlier drop in fluorescence. Each sample was analyzed in duplicate per reaction.

**Figure 12:**
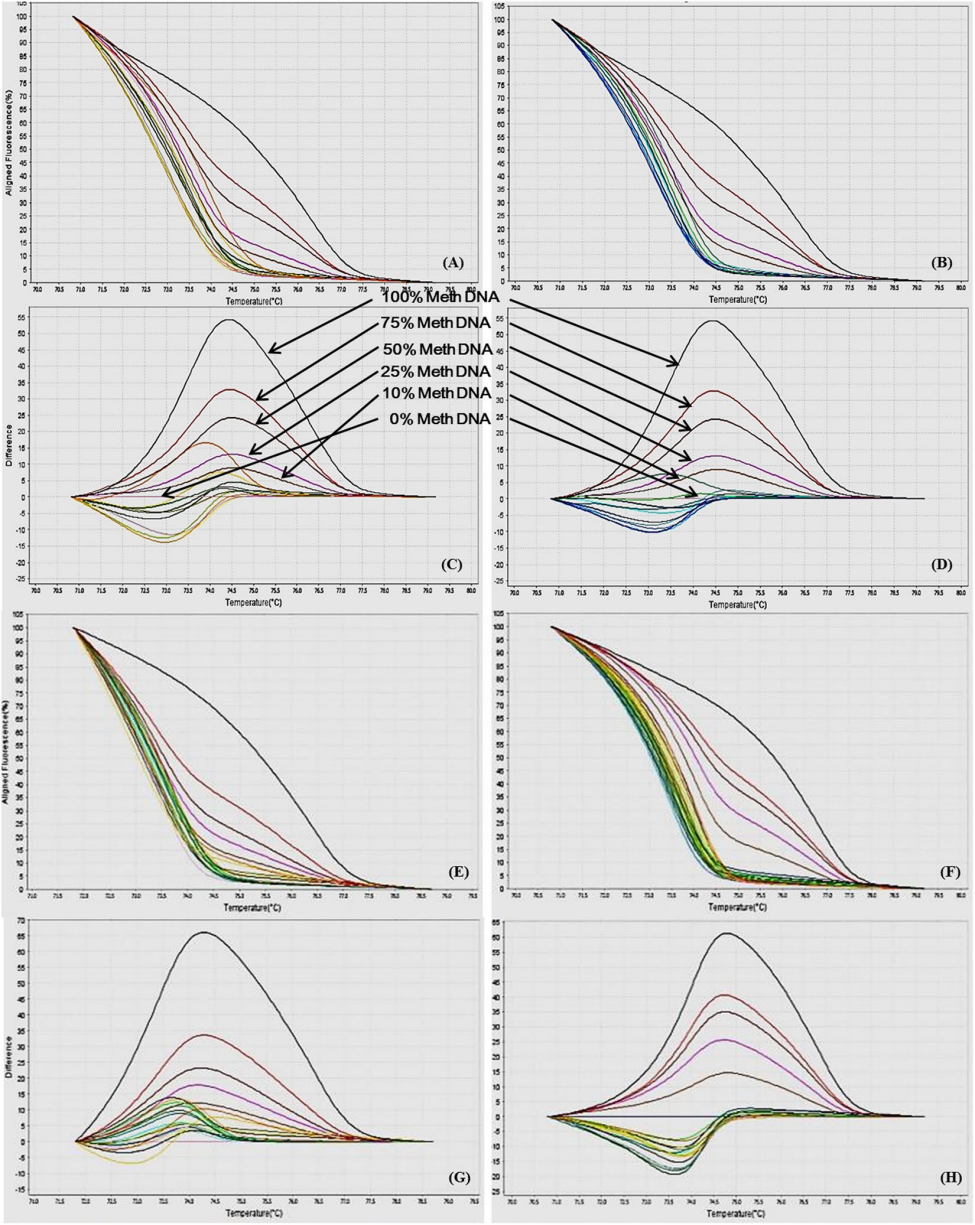
MS-HRM analysis for IL12b (**A**,**B**) Aligned melt curves and (**C**,**D**) Difference plot of 100%, 75%, 50%, 25%, 10% and 0% methylated standards, CAD patient samples (**A**,**C**, n=10) and control samples (**B**,**D**, n = 10) normalized to the 0% methylated standard DNA, showing the differential fluorescence among few representative samples of all the categories included in the study. All amplicons from the samples form a clump in the low methylation region and begin melting before the unmethylated control as can be seen by the earlier drop in fluorescence resulting from heterogeneous methylation due to heteroduplex formation. Each sample was analyzed in duplicate per reaction. (**E**,**F**) Aligned melt curves and (**G**,**H**) Difference plot of 100%, 75%, 50%, 25%, 10%, 0% methylated standards, CAD patient samples (**E**,**G**, n = 15) and control samples (**F**,**H**, n = 15) normalized to the 0% methylated standard DNA, showing the differential fluorescence of the remaining samples. All amplicons from the samples form a clump in the low methylation region and begin melting before the unmethylated control as can be seen by the earlier drop in fluorescence. Each sample was analyzed in duplicate per reaction.

**Figure 13:**
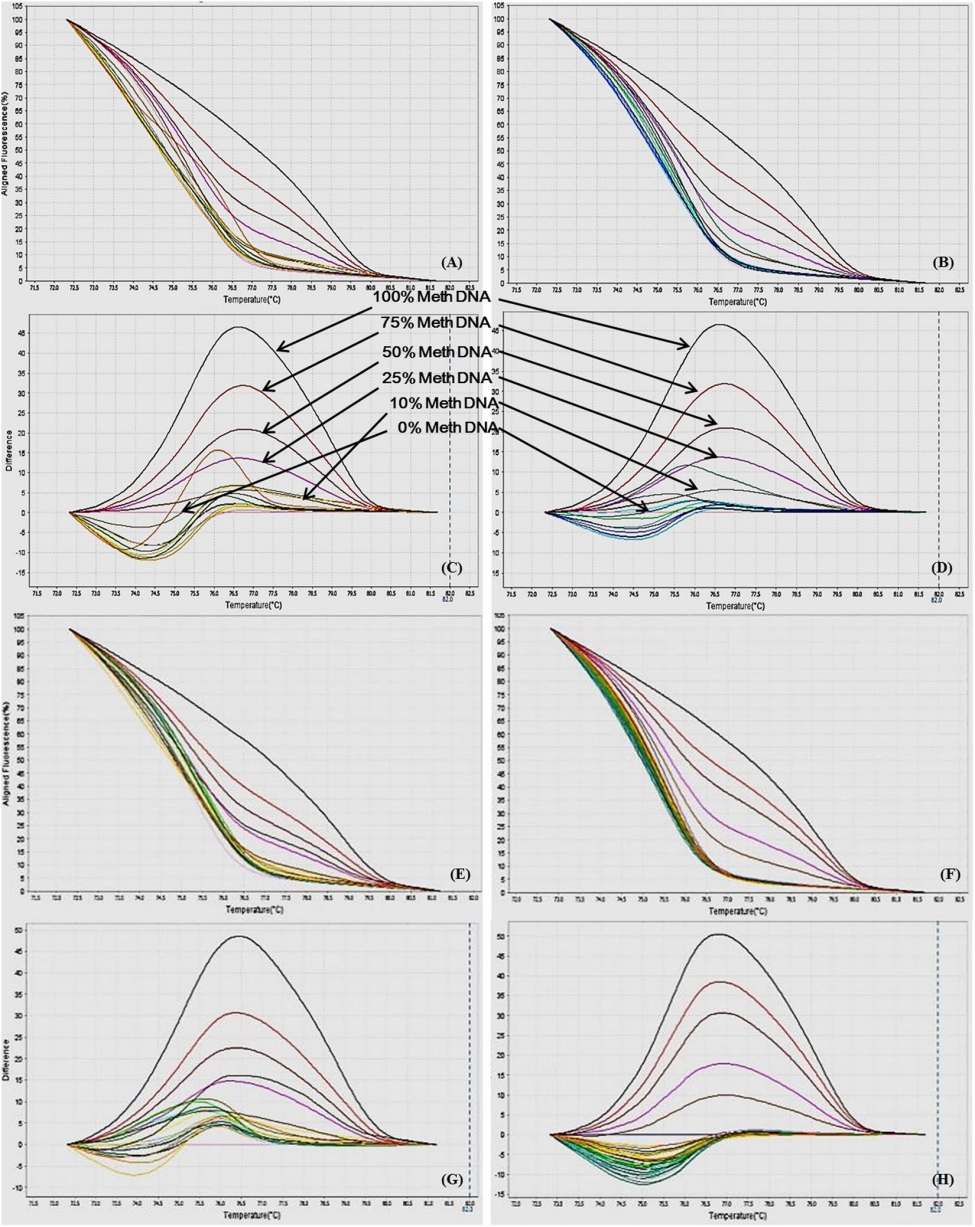
MS-HRM analysis for MHC2. (**A**,**B**) Aligned melt curves and (**C**,**D**) Difference plot of 100%, 75%, 50%, 25%, 10% and 0% methylated standards, CAD patient samples (**A**,**C**, n=10) and control samples (**B**,**D**, n = 10) normalized to the 0% methylated standard DNA, showing the differential fluorescence among few representative samples of all the categories included in the study. All amplicons from the samples form a clump in the low methylation region and begin melting before the unmethylated control as can be seen by the earlier drop in fluorescence resulting from heterogeneous methylation due to heteroduplex formation.  Each sample was analyzed in duplicate per reaction. (**E**,**F**) Aligned melt curves and (**G**,**H**) Difference plot of 100%, 75%, 50%, 25%, 10%, 0% methylated standards, CAD patient samples (**E**,**G**, n = 15) and control samples (**F**,**H**, n = 15) normalized to the 0% methylated standard DNA, showing the differential fluorescence of the remaining samples. All amplicons from the samples form a clump in the low methylation region and begin melting before the unmethylated control as can be seen by the earlier drop in fluorescence. Each sample was analyzed in duplicate per reaction.

**Figure 14:**
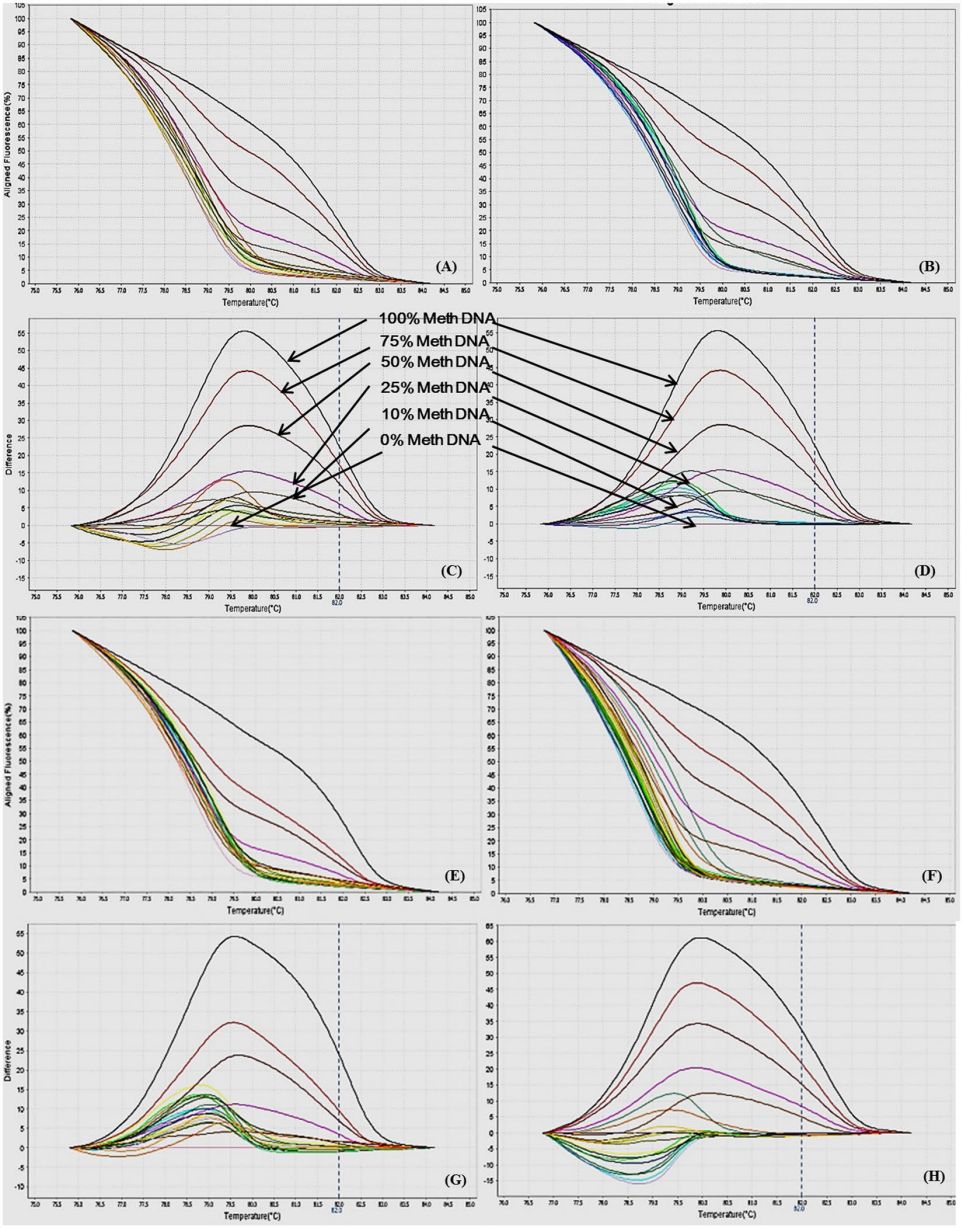
MS-HRM analysis for iNOS. (**A**,**B**) Aligned melt curves and (**C**,**D**) Difference plot of 100%, 75%, 50%, 25%, 10% and 0% methylated standards, CAD patient samples (**A**,**C**, n=10) and control samples (**B**,**D**, n = 10) normalized to the 0% methylated standard DNA, showing the differential fluorescence among few representative samples of all the categories included in the study. All amplicons from the samples form a clump in the low methylation region and begin melting before the unmethylated control as can be seen by the earlier drop in fluorescence resulting from heterogeneous methylation due to heteroduplex formation.  Each sample was analyzed in duplicate per reaction. (**E**,**F**) Aligned melt curves and (**G**,**H**) Difference plot of 100%, 75%, 50%, 25%, 10%, 0% methylated standards, CAD patient samples (**E**,**G**, n = 15) and control samples (**F**,**H**, n = 15) normalized to the 0% methylated standard DNA, showing the differential fluorescence of the remaining samples. All amplicons from the samples form a clump in the low methylation region and begin melting before the unmethylated control as can be seen by the earlier drop in fluorescence. Each sample was analyzed in duplicate per reaction.

**Figure 15:**
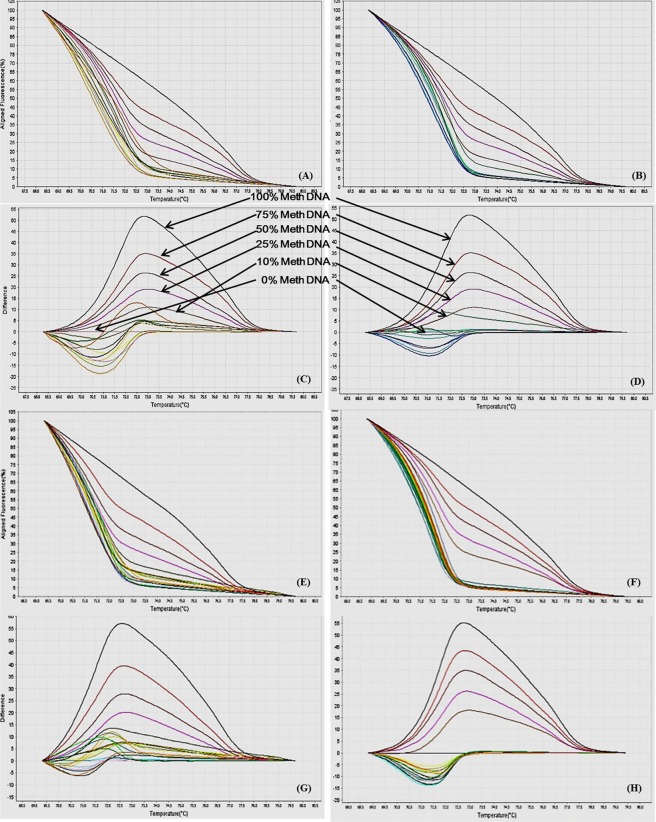
MS-HRM analysis for SOCS5. (**A**,**B**) Aligned melt curves and (**C**,**D**) Difference plot of 100%, 75%, 50%, 25%, 10% and 0% methylated standards, CAD patient samples (**A**,**C**, n=10) and control samples (**B**,**D**, n = 10) normalized to the 0% methylated standard DNA, showing the differential fluorescence among few representative samples of all the categories included in the study. All amplicons from the samples form a clump in the low methylation region and begin melting before the unmethylated control as can be seen by the earlier drop in fluorescence resulting from heterogeneous methylation due to heteroduplex formation.  Each sample was analyzed in duplicate per reaction. (**E**,**F**) Aligned melt curves and (**G**,**H**) Difference plot of 100%, 75%, 50%, 25%, 10%, 0% methylated standards, CAD patient samples (**E**,**G**, n = 15) and control samples (**F**,**H**, n = 15) normalized to the 0% methylated standard DNA, showing the differential fluorescence of the remaining samples. All amplicons from the samples form a clump in the low methylation region and begin melting before the unmethylated control as can be seen by the earlier drop in fluorescence. Each sample was analyzed in duplicate per reaction.

**Figure 16:**
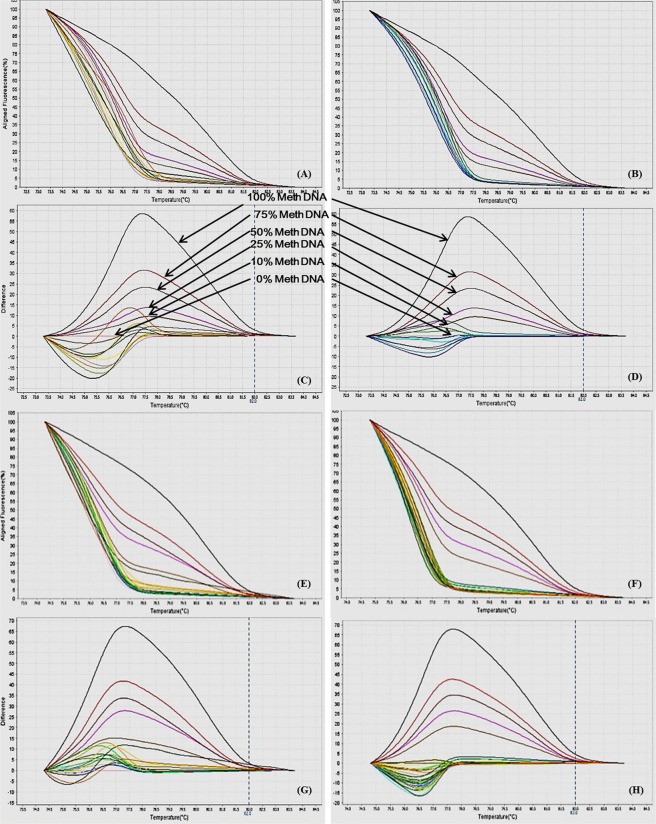
MS-HRM analysis for JAK1. (**A**,**B**) Aligned melt curves and (**C**,**D**) Difference plot of 100%, 75%, 50%, 25%, 10% and 0% methylated standards, CAD patient samples (**A**,**C**, n=10) and control samples (**B**,**D**, n=10) normalized to the 0% methylated standard DNA, showing the differential fluorescence among few representative samples of all the categories included in the study. All amplicons from the samples form a clump in the low methylation region and begin melting before the unmethylated control as can be seen by the earlier drop in fluorescence resulting from heterogeneous methylation due to heteroduplex formation.  Each sample was analyzed in duplicate per reaction. (**E**,**F**) Aligned melt curves and (**G**,**H**) Difference plot of 100%, 75%, 50%, 25%, 10%, 0% methylated standards, CAD patient samples (**E**,**G**, n = 15) and control samples (**F**,**H**, n = 15) normalized to the 0% methylated standard DNA, showing the differential fluorescence of the remaining samples. All amplicons from the samples form a clump in the low methylation region and begin melting before the unmethylated control as can be seen by the earlier drop in fluorescence. Each sample was analyzed in duplicate per reaction.

**Figure 17:**
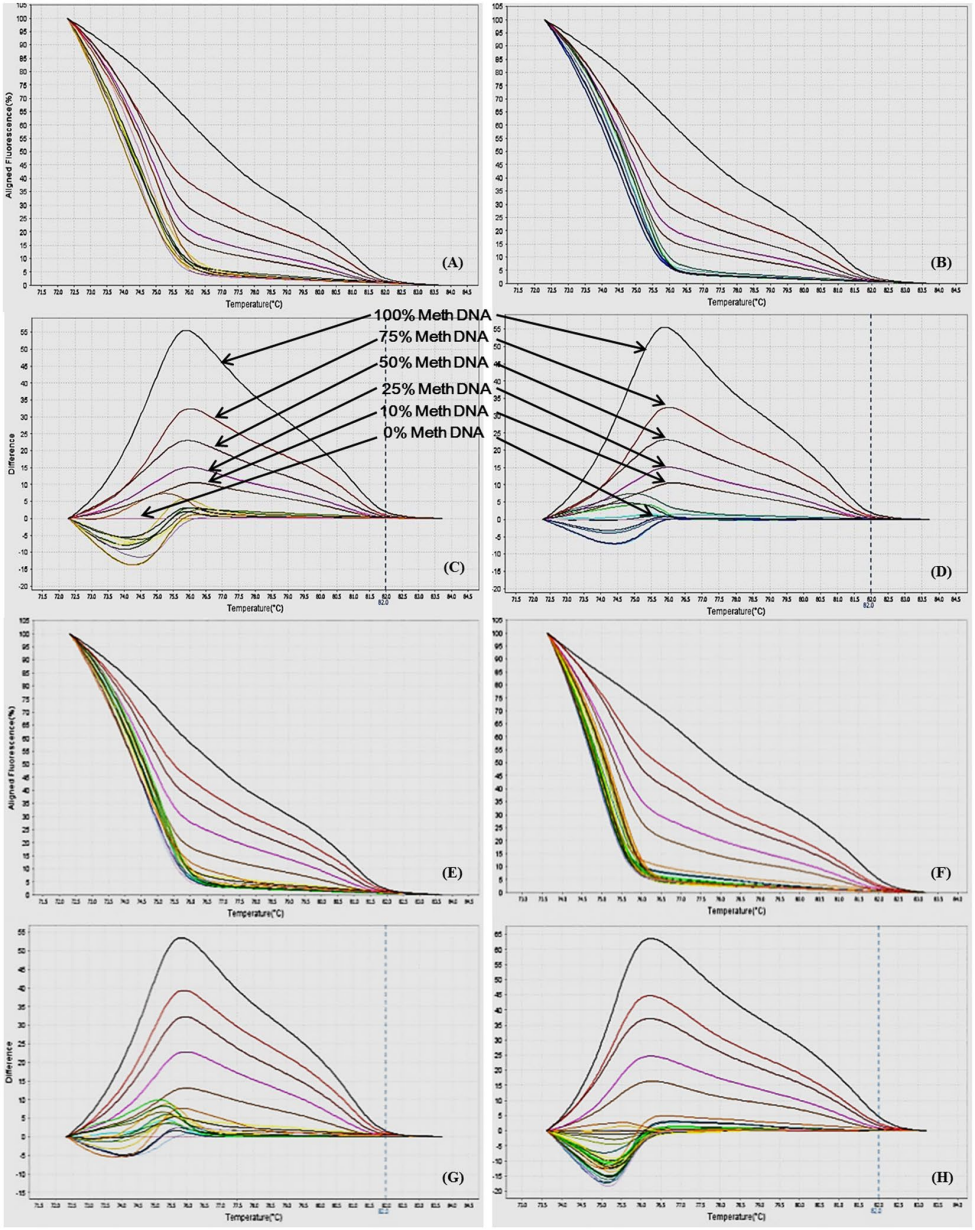
MS-HRM analysis for JAK2 promoter methylation. (**A**,**B**) Aligned melt curves and (**C**,**D**) Difference plot of 100%, 75%, 50%, 25%, 10% and 0% methylated standards, CAD patient samples (**A**,**C**, n = 10) and control samples (**B**,**D**, n = 10) normalized to the 0% methylated standard DNA, showing the differential fluorescence among few representative samples of all the categories included in the study. All amplicons from the samples form a clump in the low methylation region and begin melting before the unmethylated control as can be seen by the earlier drop in fluorescence resulting from heterogeneous methylation due to heteroduplex formation.  Each sample was analyzed in duplicate per reaction. (**E**,**F**) Aligned melt curves and (**G**,**H**) Difference plot of 100%, 75%, 50%, 25%, 10%, 0% methylated standards, CAD patient samples (**E**,**G**, n = 15) and control samples (**F**,**H**, n = 15) normalized to the 0% methylated standard DNA, showing the differential fluorescence of the remaining samples. All amplicons from the samples form a clump in the low methylation region and begin melting before the unmethylated control as can be seen by the earlier drop in fluorescence. Each sample was analyzed in duplicate per reaction.

In this study we have analyzed the gene-specific promoter CpG methylation status of various genes involved in macrophage polarization via real-time PCR based MS-HRM assay. By using the optimized MS-HRM assay, we evaluated gene-specific promoter methylation in a total of 50 genomic DNA samples isolated from PBMCs of: a) 25 CAD patients, and b) 25 healthy controls. When MS-HRM assay was performed, the promoters of *STAT1*, *STAT6*, *JAK1*, *JAK2*, *MHC2*, *iNOS*, *IL12b* and *SOCS5* were generally found to be methylated heterogeneously. Interestingly, melting profiles obtained for most of the samples including both CAD patients and controls, heterogeneous type of DNA methylation was observed as evident from their melting curves which did not conform to any of the methylation standards (Figs 10–17(A,B) and (E,F)). However, our data clearly indicates that there was a clear-cut difference in the pattern of DNA methylation status of above mentioned genes in PBMCs of CAD patients in comparison to healthy controls (Figs 10–17(C,D) and (G,H)). These complex melting curves are result of heteroduplex formation between closely associated single complementary DNA strands.

MS-HRM analysis can determine the methylation status of all CpG dinucleotides within the amplicon, but cannot determine the methylation status of individual CpG dinucleotides. However, in comparison to several other methods, MS-HRM analysis can detect heterogeneous methylation^31–33^. Aligned melt curves obtained for heterogeneously methylated templates of our samples differed in the shape from those observed for mixtures of fully methylated and unmethylated DNA standards. In addition, the melting profile of CAD patient and control samples observed in the derivative plots did not show distinct peaks as observed for fully methylated and unmethylated DNA standards.

However, the studied genes in PBMCs showed low levels of DNA methylation in both CAD patients as well as control subjects (Figs 10–17(A–H)). The melting curve of few samples did not intersect into the methylated area but showed an earlier melting as compared to the unmethylated control. This was typically due to heteroduplexes formation between sequences that had comparatively low levels of DNA methylation. Whereas, other samples had melting profiles that finished melting shortly after the unmethylated control DNA. This was consistent with low to moderate levels of DNA methylation that is also heterogeneous.

To complement the qualitative information from the melting profiles, single methylation percentage values of samples with unknown methylation were calculated to estimate the quantitative DNA methylation information. Percentage methylation was estimated using an interpolation curve which was derived by the polynomials interpolation method. For this “polyfit” interpolating function, within program MatLab was used. In each MS-HRM experiment six aligned fluorescence percentage values were calculated corresponding to each methylated standard (0%, 10%, 25%, 50%, 75% and 100%) from the aligned melt curves (Table 1).

**Table 1:** Gene specific temperature range of DNA standards used in each MS-HRM experiment for calculating the percentage methylation of unknown samples.

Gene	Temperature range (°C)
*STAT1*	73.5–80.5
*STAT6*	67.5–74.5
*IL12b*	71–77
*MHC2*	72.5–79.5
*iNOS*	76–83
*SOCS5*	68.5–75.5
*JAK1*	73.5–80.5
*JAK2*	72.5–79.5

**Figure 18:**
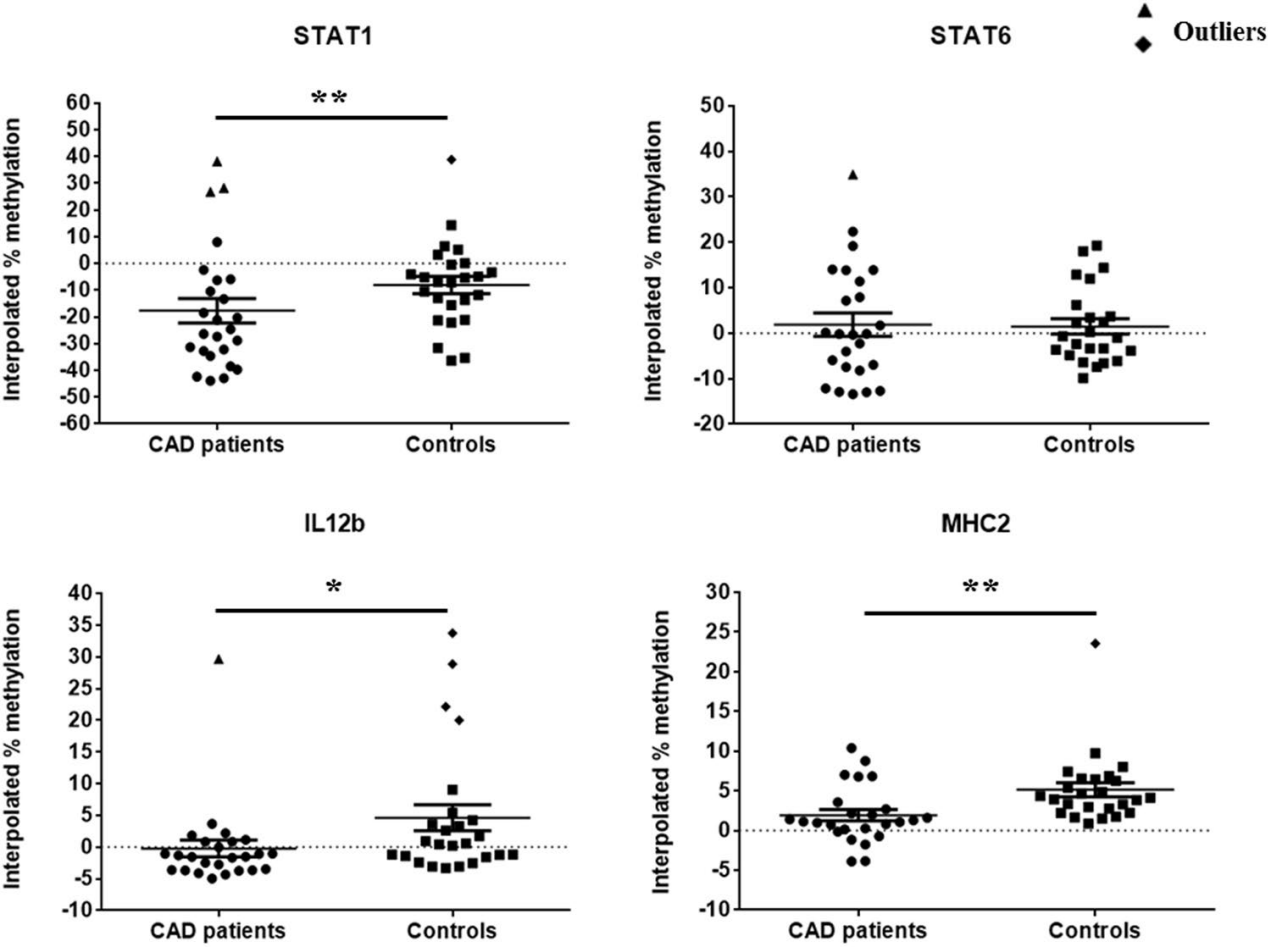
Scatter plots showing the mean percentage methylation level of STAT1, STAT6, IL12b and MHC2 promoter region, estimated for individual clinical samples including CAD patients and controls as analyzed by MS-HRM analysis (P values were estimated by two-tailed, independent t-test). *p < 0.05, **p < 0.01. Outliers were excluded from the analysis.

**Figure 19:**
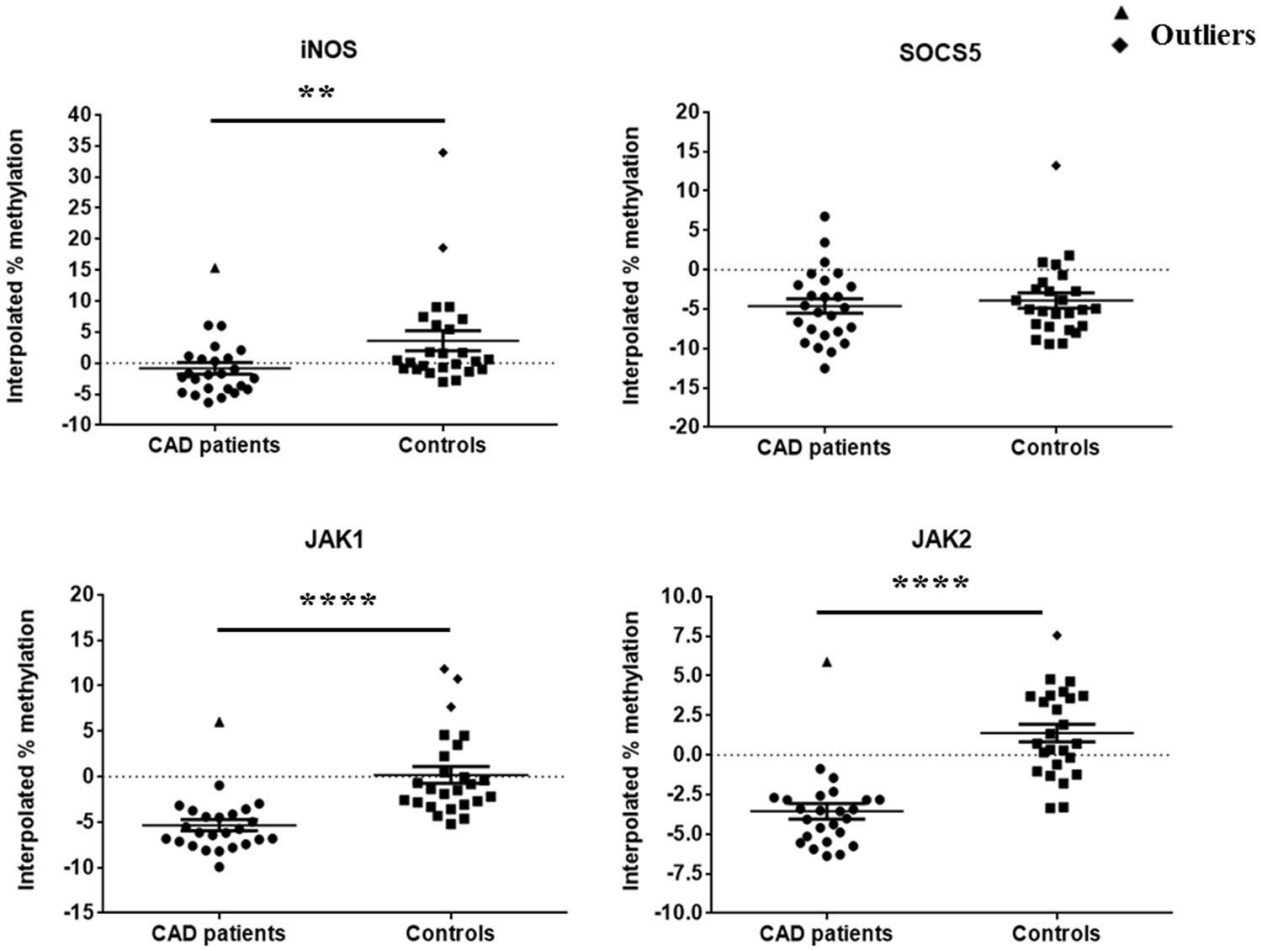
Scatter plots showing the mean percentage methylation level of iNOS, SOCS5, JAK1 and JAK2 promoter region, estimated for CAD patient and control samples as analyzed by MS-HRM analysis (P values were estimated by two-tailed, independent t-test). **p < 0.01, ****p < 0.001. Outliers were excluded from the analysis.

Methylation level of *STAT1*, *STAT6*, *IL12b*, *MHC2*, *iNOS*, *SOCS5*, *JAK1* and *JAK2* promoter by MS-HRM are shown in Figs 10–17(A–H)). It is clearly evident from the figures that the promoter region of all the genes analyzed by MS-HRM showed lower methylation in both CAD patients and controls, with methylation level not rising above 10% methylated standard DNA for most of the samples. MS-HRM analysis for gene-specific promoter methylation, revealed hypomethylation in all samples for all the genes analyzed. However, no significant difference was observed in the mean interpolated percentage methylation of *STAT6* and *SOCS5* promoter region between CAD patients and control subjects (Figs 18 and 19). MS-HRM analysis of STAT6 and SOCS5 promoter region showed mean percentage methylation of 0.4988 ± 2.229 and −4.617 ± 0.9164, respectively, in CAD patients as compared to 1.471 ± 1.699 and −4.615 ± 0.6634, respectively, in controls.

In case of *STAT1*, *IL12b*, *MHC2* and *iNOS* promoter region, a consistent hypomethylated pattern was observed with less than 10% methylation (Figs 18 and 19), except for few samples, which showed higher methylation levels than other samples in the groups. A significant difference was observed in the mean percentage methylation of *STAT1*, *IL12b*, *MHC2*, *iNOS*, *JAK1* and *JAK2* promoter region in CAD patients when compared to control subjects. The mean percentage methylation in CAD patients was found to be −24.35 ± 3.079 (p < 0.01), 1.924 ± 0.7169 (p < 0.01) and −1.524 ± 0.6976 (p < 0.01) as compared to the mean percentage methylation of −10.03 ± 2.655, 4.369 ± 0.4716 and 1.644 ± 0.7810 in control samples by MS-HRM analysis of *STAT1*, *MHC2* and *iNOS*, respectively. Whereas, MS-HRM analysis of IL12b promoter revealed the mean percentage methylation of −1.474 ± 0.4741 (p < 0.05) in CAD patients as compared to 0.5325 ± 0.7064 in control subjects (Fig. 18). Analysis of *JAK1* and *JAK2* promoter region methylation in CAD patients and control subjects also revealed lower methylation levels (Fig. 19), below 5% in both the genes; however it was significantly different in CAD patients as compared to the controls. In case of *JAK1*, mean percentage methylation in CAD patients was found to be −5.806 ± 0.4220 (p < 0.001) as compared to the mean percentage methylation of −1.173 ± 0.5974 in control samples. Whereas, in *JAK2* promoter region, mean percentage methylation in CAD patients was found to be −3.954 ± 0.3101 (p < 0.001) as compared to mean percentage methylation of 1.124 ± 0.5045 in control samples.

Table 2 shows the within-person correlations of CAD patients and controls between percentage promoter DNA methylation and clinical characteristics. The percentage DNA methylation of PBMCs showed no significant correlation with clinical characteristics in both CAD patients and controls for all of the genes analyzed by MS-HRM. However, in controls, percentage promoter DNA methylation of PBMCs exhibit strong negative associations with serum total cholesterol levels, showing Pearson correlation coefficients (r) of −0.6161 (p = 0.0029), −0.4694 (p = 0.0207) and −0.5512 (p = 0.0052), respectively, for *IL12b*, *STAT1* and *JAK2*. The percentage DNA methylation of *IL12b*, *iNOS*, *MHC2* and *SOCS5* showed negative correlation with hypertension, whereas the percentage DNA methylation of *STAT1*, *STAT6*, *JAK1* and *JAK2* showed positive correlation with hypertension in CAD patients, although non-significant.

**Table 2:** Within-person Pearson correlation coefficient of CAD patients and controls between percentage promoter DNA methylation and clinical characteristics.

Correlation (r) % DNA Meth v/s		Age	Gender	Hyper-tension	Smoking status	TC	TG	LDL-C	HDL-C
*IL12b*	CAD patients Controls	0.0312 –0.2613	−0.1218 –0.1323	−0.1019 UD (HL)	−0.021 UD (HL)	−0.2708 −0.6161** (0.0029)	−0.1786 −0.2972	−0.2693 −0.0532	−0.099 −0.3691
*iNOS*	CAD patients Controls	−0.2039 0.0770	−0.2124 −0.0080	−0.1320 UD (HL)	−0.2410 UD (HL)	0.0899 0.2598	−0.0226 0.4959	0.0235 −0.3301	0.0048 0.3097
*MHC2*	CAD patients Controls	−0.1831 −0.0368	0.0189 −0.0619	−0.3618 UD (HL)	−0.1036 UD (HL)	0.1357 −0.2754	−0.2030 0.1475	−0.1938 −0.0715	0.0956 −0.2688
*SOCS5*	CAD patients Controls	−0.0498 −0.1261	−0.1855 −0.0015	−0.1995 UD (HL)	0.0459 UD (HL)	0.0603 −0.1355	−0.1974 0.2758	−0.1875 −0.1902	0.2773 0.1224
*STAT1*	CAD patients Controls	0.0214 −0.2127	−0.0891 0.0246	0.2765 UD (HL)	0.0648 UD (HL)	0.0336 −0.4694* (0.0207)	0.2456 −0.0187	−0.1321 −0.095	0.2095 −0.3911
*STAT6*	CAD patients Controls	−0.0356 −0.1092	−0.0711 −0.3123	0.1639 UD (HL)	0.1814 UD (HL)	0.0611 −0.250	0.0357 −0.0188	−0.0742 −0.2502	0.1755 0.0150
*JAK1*	CAD patients Controls	0.0377 0.0927	−0.1190 −0.1425	0.1525 UD (HL)	−0.2229 UD (HL)	−0.0043 −0.1892	−0.0428 0.1843	0.0661 −0.1163	−0.2357 −0.2325
*JAK2*	CAD patients Controls	−0.1677 −0.0673	−0.1776 −0.2261	0.0591 UD (HL)	0.1171 UD (HL)	−0.1071 −0.5512** (0.0052)	−0.0408 −0.1569	−0.1429 −0.1161	−0.0278 −0.2622

r correlation coefficient, UD undetermined, HL horizontal line, *p < 0.05, **p < 0.01.

## Discussion

Atherosclerosis ensues as a consequence of endothelial dysfunction leading to monocyte migration into the sub-endothelial intima and differentiation into macrophages, which accumulate oxidized-low density lipoprotein (ox-LDL) and transform into foam cells^34,35^. The transcriptional regulation of macrophage differentiation and polarization has been extensively studied. For instance, the transcription factors PU.1 and C/EBP play a crucial role in the development of macrophages^6,36^. Recently, it has been demonstrated that macrophages can exist as mixed M1/M2 phenotypes *in vivo*, especially in complex pathological settings such as atherosclerosis^6^. Even though, it remains unknown if such heterogeneity is due to mixed polarization of individual cells or coexistence of macrophages with distinct phenotypes^6,12^. M1/M2 macrophages are potentially regulated by genetic or epigenetic background in human individuals and have been implicated in plaque destabilization^37^. These findings indicate a new way to reduce CVD risk by balancing the M1/M2 polarization during atherosclerosis.

Epigenetics refers to the phenomena that govern how information encoded in DNA is expressed in a tissue- and context-specific manner without altering the actual genetic code^38^. Numerous studies have explored the role of epigenetic modifications in gene expression associated with atherosclerosis and inflammation^39–41^. However, due to the dynamic nature and tissue heterogeneity of atherosclerotic disease, not much is known about the precise role of DNA methylation involved in the modulation of inflammatory and anti-inflammatory genes during atherogenesis^41^. Current literature suggests that global DNA hypermethylation is linked to inflammation and is associated with higher mortality in atherosclerosis-related diseases^42^. In one study, promoter hypomethylation of the Toll-like receptor (TLR) 2 gene was found to be related with enhanced pro-inflammatory responses^43^. Castro *et al*., (2003) found that patients with vascular disease had significantly lower genomic DNA methylation using the intracellular S-adenosylmethionine/S-adenosylhomocysteine (SAM/SAH) ratio as a predictor of cellular methylation capacity^44^. In a population-based prospective study of Singaporean Chinese, the link between prevalence of CVD (MI, stroke) and its predisposing conditions (hypertension, diabetes) and global genomic DNA methylation in peripheral blood leukocytes, as represented by ALU and Satellite 2 (AS) repetitive element DNA methylation, were examined^25^. The male study subjects with a diagnosis of CVD or its predisposing conditions had the highest global DNA methylation level at baseline while the male study subjects, who were free of CVD/predisposing conditions at follow-up, had the lowest ones. The results suggested that men demonstrated significantly higher levels of global DNA methylation than women which were positively associated with the prevalence of CVD or its predisposing conditions^25^.

Although, variations of global DNA methylation in PBMCs have been explored in CAD by several groups, the results remain contradictive. For instance, early studies stated both positive and negative associations between CAD and global DNA methylation measured in LINE-1 and ALU repeats^22–26^. Baccarelli *et al*. (2010) found a correlation between blood LINE-1 hypomethylation and baseline ischemic heart diseases and stroke in the Boston-area Normative Aging Study^22^. Haley L *et al*. (2011) reported a correlation between higher levels of LDL-C and LINE-1 hypomethylation in a Samoan islander population^23^. In a prospective study led by Guarrera *et al*. (2015), global DNA hypomethylation measured in LINE-1 repeats was found to be associated with CVD and MI risk in men^24^. In contrast, elevated global DNA methylation in CAD has also been observed in some studies^25,26^. These studies utilized different detection methods on different populations, thus there is still an ambiguity about the comparability and extent to which these repeat sequences reflect global DNA methylation content^45^. Recently, it has been reported that external stimuli, such as lipid particles can prime circulating immune cells to attain a long-term epigenetic memory^46^. In one of the studies it was observed that the TG-lowering drug fenofibrate was not able to significantly reverse DNA methylation changes associated with lipids, after a 3-week daily treatment^47^. This suggests that these changes occurred early in hematopoietic stem cells, where lipid priming has been initiated but did not precipitate in circulatory levels or were not evident in circulation.

The reasons leading to the contrary results are not known explicitly, but could be related to the following factors. Firstly, DNA methylation status may be related with different stages of CVDs. Atherogenic lipoprotein-induced DNA hypermethylation before the initiation of atherosclerosis suggests that aberrations in DNA methylation are amongst the earliest cellular alterations in atherosclerosis. Secondly, different biomarkers, represented as global methylation status, were used; as well as different samples including different organs, cell types, and tissues were examined in each study. Thirdly, different analytical methods were applied to assess the methylation status in these studies. Since each method had a different sensitivity or specificity as well as limitations, it is inevitable that some false positives or negatives were observed in these results.

Methylation status of the promoter region can be investigated using various experimental methodologies, which includes PCR-based methods such as Combined Bisulfite Restriction Analysis (COBRA), MethyLight-PCR and pyrosequencing^48–51^. The COBRA assay involves amplification followed by restriction endonuclease digestion of bisulfite-modified DNA. However, the need for specific restriction enzyme recognition sequences within the target region is a major limitation of this method^48^. Drawbacks of using MethyLight, a quantitative real-time Taqman-based assay impose the necessity to use more expensive Taqman dual-labelled hydrolysis probes along with a reference normalization assay^49^. On the other hand, pyrosequencing indicates the methylation status of individual CpG dinucleotide within a small target region, although it also entails the use of specialized equipment^50,51^. In addition, pyrosequencing does not provide information regarding the methylation status of each site with respect to the other CpG site on the same DNA strand^50,51^. Hence subpopulations of sequences which have very different methylation patterns cannot be differentiated. On the contrary, HRM-PCR is considered as a robust method for estimating methylation levels^32,52^. HRM utilizes the characteristic shape of the melting curve of bisulfite converted DNA sample to assess methylation level when compared to the samples with known methylation levels^53^. MS-HRM method can rapidly assess the presence of DNA methylation and at the same time can also distinguish homogeneous methylation from heterogeneous methylation patterns. MS-HRM permits prompt identification of heterogeneously methylated DNA samples, depending on a characteristic complex melting profile which do not lie within the methylation range as defined by unmethylated and methylated DNA standards.

In the present study, our aim was to compare gene-specific promoter DNA methylation status of macrophage polarization genes in PBMCs between CAD cases and controls. Most of the samples including both CAD patients and control subjects in our MS-HRM analysis showed complex melting pattern which is characteristic of heterogeneous type of DNA methylation. Importantly, promoter DNA methylation of M1 macrophage genes was significantly altered in cases as compared to controls. There was indeed a clear-cut variation in the pattern of gene-specific promoter CpG methylation status of *STAT1*, *STAT6*, *IL12b*, *MHC2*, *iNOS*, *SOCS5*, *JAK1* and *JAK2* between CAD patients versus healthy controls. These differences are clearly evident from the respective aligned melt curves and difference plot of each gene showing a complex melting pattern assessed by MS-HRM assay. DNA methylation is a dynamic process and a heritable epigenetic element related to demographical, clinical as well as environmental factors, which are strong cofounding variables contributing to the measured phenotype. However, in the current study, only age, gender, hypertension, blood lipids and smoking habits have been taken into account, and characteristics such as PBMC counts and classifications were not included, which remains one of the limitations. Since, the mixed composition of various cells within the peripheral blood has an influence on the global DNA methylation, it is possible that it may also affect gene-specific promoter DNA methylation^54–57^. But, whether the varied DNA methylation is the consequence of PBMCs classification, or the cause, or the company, needs to be further explored.

The reason for heterogeneous methylation observed could be that not all CpG islands in the promoter region will have complete demethylation at every CpG that will result in a heterogeneous pattern of methylation at any individual CpG dinucleotide^31,58^. The mechanism of gene silencing by heterogeneous methylation is still not known. Though, a hypothesis has been proposed which states that heterogeneous methylation may be a “passenger” that impedes with the transcription processes^59^, and thus heterogeneous methylation may play a vital role in atherogenesis. Promoters containing CpG islands can be rapidly activated in response to external stimuli because of the pre-bound RNA polymerase II and the lack of a nucleosomal barrier to the recruitment of transcription factors activated by stimulation (such as NF-κB, AP1 and IRFs)^60,61^. Moreover, data from whole genome methylation sequencing of PBMCs support the fact that CpG sites within the regions of high density such as CpG island and 5′-UTR, were oftently unmethylated, whereas CpG sites located in introns, 3′-UTRs and repetitive elements were methylated^62^.

Surprisingly, differences in promoter DNA methylation of analysed genes may not affect gene expression levels for the reason that differences in gene-specific promoter DNA methylation do not appear to be associated with differences in gene expression levels, which we did not test in our current study. However, it suggests that gene expression levels are influenced by other regulatory mechanisms which are predominant over DNA methylation. One of the possibilities is that differences in promoter DNA methylation of genes, poise them for expression in response to external stimuli. In addition, it is also unlikely to decide whether the single nucleotide polymorphisms (SNPs) cause variation in promoter DNA methylation (affecting the recruitment of histone protein modifying enzymes) or whether the SNPs cause changes in specific histone modifications that favor or hinder promoter DNA methylation. An additional possibility remains that the promoter methylation status affects other gene features such as alternative transcript initiation or splicing, which may be true for the subset of intragenic differentially methylated regions (DMRs). Since, gene promoter regions are underrepresented among the DMRs, thus further investigations are required by additional comprehensive molecular studies.

The MS-HRM assay has the ability to qualitatively measure the changes in DNA methylation pattern where methylation of individual CpG site or mean percentage methylation is not warranted. The assay provides a useful technique for the ongoing investigation in search of a potential biomarker of the transcriptional control. Heterogeneous methylation produce complex melt curves which cannot be simply quantified by comparison to melt curves of unmethylated and methylated control DNA. To quantify a single percentage methylation value of samples with unknown methylation, percentage methylation was estimated using an interpolation curve which was derived using the method of interpolation of polynomials^63^. For this, within the program MatLab, the “polyfit” interpolating function was used. Moreover, we suggest that the MS-HRM assay is a rapid and inexpensive screening method for the identification as well as investigation of minor but potential disease-initiating aberrant gene-specific promoter DNA methylation changes in atherosclerosis.

In conclusion, epigenetic regulation in atherosclerosis is still an emerging field that is gradually understood by the people and needs to be enriched. It is evident from the correlations between atherosclerosis and global DNA methylation, that epigenetics does play a crucial role in atherogenesis; however, it remains unknown which genes are under direct regulation by DNA methylation. In addition, DNA methylation studies apart from expanding our knowledge of atherosclerotic mechanisms, may pave a way for therapies in form of “epigenetic drugs,” such as DNA methyltransferase (DNMT) inhibitors and diagnostic biomarkers. Since monocytes as well as macrophages play a significant role in atherosclerosis, epigenetic markers including gene-specific promoter DNA methylation based on monocyte/macrophage might aid as diagnostic markers or drug targets for clinical application. Although, there is a plethora of studies in finding clinical epigenetic biomarkers and drugs, further efforts are still required to clarify the full therapeutic potential of the drugs targeting specific epigenetic mechanisms in monocyte/macrophage as well as M1/M2 macrophage polarization during atherosclerosis. Since, M1/M2 polarization is a critical factor for plaque stability, skewing the M1/M2 macrophage balance towards a more preferable phenotype using novel agents may help in curbing atherosclerotic disease. Therefore, epigenetic gene regulation by DNA methylation-related interventions may open new vistas for atherosclerotic disease management in the future.

## Materials and Methods

### Study participants

The permission for this study was obtained from the Institutional Ethics Committee (IEC) of the Post Graduate Institute of Medical Education and Research (PGIMER), Chandigarh, India (IEC ref. no. INT/IEC/2015/251, dated 15/07/2015) and all experiments were performed according to the relevant guidelines and regulations in the Department of Experimental Medicine and Biotechnology in collaboration with the Department of Cardiology, PGIMER. A total number of 25 stable CAD patients and 25 healthy individuals, both male and female, aged between 18–65 years attending the Cardiology Clinic at the Advanced Cardiac Centre, PGIMER were enrolled. A fully informed consent was also obtained from the study subjects prior to their participation in the study. The subjects with >50% stenosis of one major coronary artery, confirmed by angiography were included as CAD patients in the study. Exclusion criteria for CAD patients included past or present history of diabetes mellitus, chronic liver disease, chronic renal failure, acute heart failure, severe non-coronary CVDs, coronary artery spasm, cardiomyopathy, congenital heart disease, systemic infections or any other inflammatory diseases, and the use of immunosuppressant or chemotherapeutic agents. The recruited patients with stable CAD were on their regular medications which included either statins or angiotensin converting enzyme (ACE) inhibitors or β-blockers or calcium channel blockers. At no time point the conventional drug treatment given to CAD patients was stopped. Thus, this study had no adverse effect on survival and health of the study subjects. Healthy, unrelated subjects of both sexes, belonging to the same age group, ethnic origin and socioeconomic background were included as controls. Control subjects were selected from the individuals attending the clinics at PGIMER, relatives accompanying the patients at outdoor patient clinics and volunteers from the staff of the PGIMER. Healthy individuals with a normal angiogram were included as controls (non-CAD). Subjects with past or present history of CAD, hypertension, diabetes mellitus and any other inflammatory or infectious disease were excluded from the study. The characteristics, including age, gender, systolic blood pressure (SBP), diastolic blood pressure (DBP), heart rate, hypertension and smoking status as well as their serum lipid and lipoprotein profile at the time of recruitment of the study subjects are summarized in Table 3.

**Table 3:** Characteristics of patients with CAD and control subjects.

Characteristic	Cases (n = 25)	Controls (n = 25)	p-value
Age (y)	52.12 ± 1.513	49.80 ± 1.708	ns (0.3143)^a^
Gender (M/F)	M = 22 (88%)	M = 20 (80%)	ns (0.7019)^b^
	F = 3 (12%)	F = 5 (20%)	
SBP (mmHg)	115.68 ± 3.091	122 ± 1.601	ns (0.0778)^a^
DBP (mmHg)	76.08 ± 1.643	81.08 ± 1.359	*(0.0234)^a^
HR (/min)	83.40 ± 2.411	80.08 ± 1.160	ns (0.2231)^a^
Hypertension	Yes = 18 (72%)	Yes = 0 (0%)	***(<0.0001)^b^
	No = 7 (28%)	No = 25 (100%)	
Smoker	Yes = 7 (28%)	Yes = 0 (0%)	**(0.0096)^b^
	No = 18 (72%)	No = 25 (100%)	
TC (mg/dl)	135.72 ± 4.533	138.80 ± 3.857	ns (0.6073)^a^
Triglyceride (mg/dl)	135.76 ± 3.821	115.20 ± 3.653	***(0.0003)^a^
LDL-C (mg/dl)	66.48 ± 4.533	76.20 ± 3.200	ns (0.0869)^a^
HDL-C (mg/dl)	42.00 ± 1.591	48.08 ± 1.439	**(0.0067)^a^

y years, M male, F female, SBP systolic blood pressure, DBP diastolic blood pressure, HR heart rate, LDL-C low density lipoprotein-cholesterol, HDL-C high density lipoprotein-cholesterol, TC total cholesterol, ^a^Independent t-test, ^b^fisher’s exact test, ns non-significant, *p < 0.05, **p < 0.01, ***p < 0.005.

### Isolation of peripheral blood mononuclear cells (PBMCs)

5 ml blood sample was collected in ethylenediaminetetra acetic acid (EDTA) vials (BD Biosciences, USA) from each study subject through an antecubital vein (phlebotomy) after an overnight fast. PBMCs were isolated from the blood using Histopaque-1077 (Sigma-Aldrich, St. Louis, MO) density gradient centrifugation method^64^. Briefly, blood was carefully overlaid on histopaque and centrifuged at 1200 rpm for 30 minutes at room temperature. The dense buffy coat obtained after centrifugation was gently pipetted out and cells were rinsed twice with phosphate buffered saline (PBS) (pH 7.4) at 1000 rpm for 10 minutes at room temperature. Finally, the cell pellet was resuspended in fresh PBS.

### Genomic DNA extraction from PBMCs

Genomic DNA from the PBMCs of the study subjects was isolated by traditional phenol/chloroform extraction method^65^. Briefly, cells were isolated and rinsed once with PBS (pH 7.4). Cells were then incubated in lysis buffer with RNase at 37 °C for 1 h followed by incubation with Proteinase K at 56 °C for overnight. Then phenol: chloroform: isoamyl alcohol (25:24:1) was added and samples were centrifuged at 12,000 g for 10 minutes. Carefully upper aqueous layer was collected and DNA was precipitated by adding iso-propyl alcohol. The extracted DNA was dissolved in 1x TE buffer and stored at −80 °C till further analysis. DNA concentration was quantified using spectrophotometer (NanoDrop ND-1000 Spectrometer, Thermo Scientific, USA) and DNA quality was assesed by the ratio of absorbance at wavelength of 260 and 280 nm.

### Bisulfite modification of genomic DNA

The genomic DNA isolated from PBMCs was subjected to bisulfite conversion using the EpiTect Plus DNA Bisulfite Kit (Qiagen, Germany) and each step was performed according to the instructions of manufacturer. Briefly, 500 ng of DNA from each sample was treated with sodium bisulfite mix which converts all unmethylated cytosines into uracil, whilst methylated cytosines remain unchanged. Chemical modification of cytosine leads to a change in primary DNA sequence that permits detection of unmethylated cytosines from 5-methyl-cytosine. Finally, DNA was eluted in the elution buffer and stored at −20 °C till subsequent experimentation.

### Methylation Sensitive-High Resolution Melting (MS-HRM) analysis

MS-HRM is a simple and sensitive method for analyzing the methylation status by comparing the melting profiles of unknown samples to profiles from DNA with known methylation levels (standards). In order to precisely measure the percentage DNA methylation of unknown samples, a standard curve was established for each gene.

#### Methylation standards

Human fully methylated (100%) and unmethylated (0%) control DNA (EpiTect human control DNA, bisulfite converted, Qiagen, Germany) were used as standards. To produce a series of methylated and unmethylated DNA standards, 0% methylated and 100% methylated (unmethylated) DNA of equal concentration were mixed in different ratios i.e. 0%, 10%, 25%, 50%, 75% and 100% to mimic DNA samples with known levels of DNA methylation. Standard curves with known methylation levels were included in every assay and were used to estimate the percentage methylation in the promoter region of the genes analyzed.

#### Primer designing

Forward and reverse primers for MS-HRM analysis were designed according to the criteria of Wojdacz *et al*.^50^ in order to minimize PCR bias. Briefly, primers were designed with the following conditions: i) Primers should contain a limited number of CpG dinucleotides, usually 1 or upto 2 and included CpG should be as close as possible to the 5′ end of each primer, ii) One or more natural thymidine nucleotides (T) originating from a non-CpG cytosine should be included at or near the 3′end of each primer and iii) The melting temperature of the primers should be matched, preferably within 1°C. Selected primers were designed to amplify the region of interest within the promoter region of each gene using various online softwares (Table 4). Broadly following steps were performed: Promoter identification of different genes was done by online available software like http://cagt.bu.edu/page/Promoser_submit and http://rulai.cshl.edu/cgi-bin/TRED/ tred.cgi?process = searchPromForm using the GenBank accession number as provided by reference sequence database of NCBI. In-silico check of sequence specificity was carried out using Basic Local Alignment Search Tool (BLAST) of NCBI http://blast.ncbi.nlm.nih.gov/Blast.cgi.

**Table 4:** Primer sequences of genes and respective MS-HRM conditions.

Gene	Gene ID (Accession)	Annealing temperature (°C)	No of CpGs in the amplicon	Amplicon size (bp)
***STAT1*** F: 5′AAGATATGTAAATAGAACGTTAGTTTTTAG3′ R: 5′TCAACCAATTAAACGCGACTATTC3′	6772 (NG_008294)	58	19	174
***STAT6*** F: 5′GGTTGTTGTAGACGTTGAGATTTTTTA3′ R: 5′CCCGAATCCTCTAATTATAAAACCAC3′	6778 (NG_021272)	60	11	168
***IL12b*** F: 5′GGAAGTGTGCGGTTGGGAAGTTT3′ R: 5′CCTCCGCCCCTACGATAAAAAAAATT3′	3593 (NG_009618)	60	14	165
***MHC2*** F: 5′TGGGCGGGTGGTAGGAAAGTT3′ R: 5′CCCTAATCCAAAATCTAACGTACAAAAA3′	4261 (NG_009628)	60	15	165
***iNOS*** F: 5′GTTGCGGGTTTTTGGGTGTTTG3′ R: 5′CTTTATCGCTCGAAACCTACAAC3′	4843 (NG_011470)	60	17	155
***SOCS5*** F: 5′GTCGTAGTTGTTAGATTTTAAAATGGTA3′ R: 5′CCAATCCGAAAACGAATAAATAAAAAC3′	9655 (NC_000002)	56	16	137
***JAK1*** F: 5′GTCGCGGAGTATTTTGGAGTTGTAGAT3′ R: 5′CCCCGTCGCTACGCTAACTAAAAT3′	3716 (NG_023402)	60	21	162
***JAK2*** F: 5′CGAGAGGTAGTTGTTTTGCGTGGTT3′ R: 5′ACTCAACCTCCGGATTAACCTACAAT3′	3717 (NG_009904)	60	20	160

#### Polymerase chain reaction (PCR) and MS-HRM optimization

Bisulfite converted DNA was used for MS-HRM analysis. The aim of optimization was to identify the best PCR primer pair and PCR cycling conditions for the analysis of differentially methylated region of interest. PCR amplification and HRM analysis of bisulfite converted DNA was done using StepOne Plus Real-Time PCR system (Applied Biosystems, Life Technologies). A 20 μL reaction mix consisted of 20 ng of bisulphite modified control DNA standards and a final concentration of: 1x MeltDoctor HRM Master Mix (Applied Biosystems, Life Technologies), 0.25 μM forward primer, 0.25 μM reverse primer and deionized water. Cycling conditions were as follows: 95 °C for 10 min followed by 45 cycles of 95 °C for 15 sec, annealing / extension temperature for 60 sec. Analysis of melt curve occurred from 60 °C to 95 °C. HRM analysis was executed at the temperature ramping and florescence acquisition setting as recommended by the manufacturer. Analysis was done with High-Resolution Melt Software v2.0 (Applied Biosystems, Life Technologies). All participant DNA samples were analyzed in duplicate per PCR reaction on a 96-well plate which also included a set of reference methylated DNA standards, a no-template control (NTC) and a negative control (unconverted unmethylated DNA).

### Derivation of single estimates of methylation of unknown samples using MS-HRM

Single methylation percentage values of samples with unknown methylation were calculated from aligned melt curves, using the method of Migheli *et al*.^63^. In each MS-HRM experiment, six aligned fluorescence percentage values were calculated corresponding to each methylated standard (0%, 10%, 25%, 50%, 75% and 100%) from the aligned melt curves. Each aligned fluorescence percentage unit was calculated as an average of fluorescence values of the temperatures relative to the melting status in a specific temperature range for a single methylation DNA standard, respective of MS-HRM experiment of each gene. Using this data set, an interpolation curve was derived using the method of interpolation of polynomials. For this, within the program MatLab, the “polyfit” interpolating function was used (The MathWorks, Inc., USA). After obtaining the interpolation curve of the standards in an experiment, imputation of the observed average aligned fluorescence percentage for each sample was used to get its precise percentage of methylation.

### Statistical analysis

The statistical analysis between the two groups was done using two-tailed independent t-test with Welch’s correction to analyze the significance of difference between two variables. The correlation between gene-specific percentage DNA methylation and age, TC, TG, LDL-C, HDL-C, hypertension, smoking status and gender was analyzed using Pearson correlation. In all of the analysis the value of p < 0.05 was considered significant. All means are displayed with error bars represented as mean ± standard error of mean (SEM). For estimation of gene-specific mean percentage methylation of patient and control samples by interpolation, outliers were excluded from the analysis.

## Supplementary information

Supplementary figures

## Data Availability

Authors agree to make materials, data and associated protocols promptly available to readers.
