# Supplementary material for: Aberrant DNA methylation of M1-macrophage genes in coronary artery disease
Source: Sci Rep. 2019 Feb 5;9:1429. doi: 10.1038/s41598-018-38040-1 (PMC6363807; doi:10.1038/s41598-018-38040-1)
Supplement: Supplementary file 1 — Supplementary figures [file 41598_2018_38040_MOESM1_ESM.pdf]

Aberrant DNA methylation of M1-macrophage genes in coronary artery disease

Chetan Bakshi[1], Rajesh Vijayvergiya[2], Veena Dhawan[1,*]

[1]Department of Experimental Medicine and Biotechnology, Postgraduate Institute of Medical Education and Research, Chandigarh, India - 160012

[2]Department of Cardiology, Postgraduate Institute of Medical Education and Research, Chandigarh, India - 160012

***Corresponding author:**

Veena Dhawan, Professor, Room no. 2014, 2nd Floor, Department of Experimental Medicine and Biotechnology, Research Block-B, Postgraduate Institute of Medical Education and Research, Chandigarh - 160012, India.

E-mail: officialveenapgi@gmail.com

Supplementary Figures

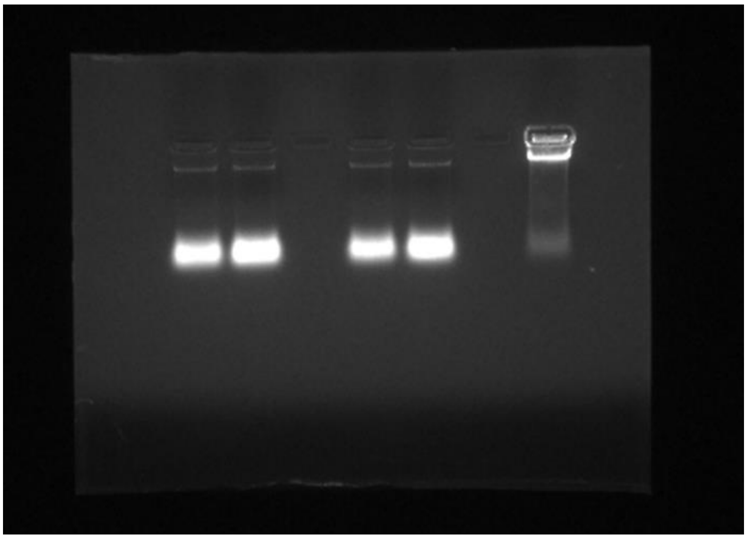

Full unedited gel for Figure 1A: Agarose gel electrophoresis of genomic DNA isolated from PBMCs of the study subjects. Lane 1 and 2 represent genomic DNA sample from CAD patients. Lane 4 and 5 represent genomic DNA sample from control subjects. Lane 7 represent genomic DNA isolated from THP-1 cells.

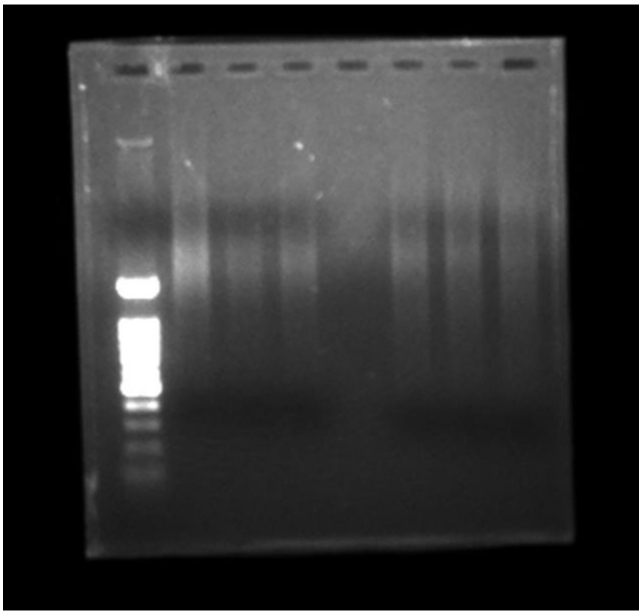

Full unedited gel for Figure 1B: Agarose gel electrophoresis of bisulphite-converted genomic DNA. Lane 2, 3 and 4 represent bisulphite-modified DNA of CAD patients. Lane 6, 7 and 8 represent bisulphite-modified DNA of control subjects. Lane 1 represents 100 bp DNA ladder.
